# Facile Synthesis of Magnetic Photocatalyst Ag/BiVO_4_/Mn_1−x_Zn_x_Fe_2_O_4_ and Its Highly Visible-Light-Driven Photocatalytic Activity

**DOI:** 10.3390/ma11050810

**Published:** 2018-05-16

**Authors:** Taiping Xie, Hui Li, Chenglun Liu, Longjun Xu

**Affiliations:** 1 Chongqing Key Laboratory of Extraordinary Bond Engineering and Advanced Materials Technology (EBEAM), Yangtze Normal University, Chongqing 408100, China; deartaiping@163.com; 2 State Key Laboratory of Coal Mine Disaster Dynamics and Control, Chongqing University, Chongqing 400044, China; 3 College of Chemistry and Chemical Engineering, Chongqing University, Chongqing 401331, China; lihui@163.com

**Keywords:** Ag/BiVO_4_/Mn_1−x_Zn_x_Fe_2_O_4_, photocatalytic activity, magnetic property, wastewater treatment, reaction mechanism

## Abstract

Ag/BiVO_4_/Mn_1−x_Zn_x_Fe_2_O_4_ was synthesized with a dip-calcination in situ synthesis method. This work was hoped to provide a simple method to synthesis three-phase composite. The phase structure, optical properties and magnetic feature were confirmed by X-ray diffraction (XRD), Fourier transform infrared spectroscopy (FTIR), X-ray photoelectron spectrometer (XPS), transmission electron microscopy (TEM), ultraviolet-visible diffuse reflectance spectrophotometer (UV-vis DRS), and vibrating sample magnetometer (VSM). The photocatalytic activity was investigated by Rhodamine B (RhB) photo-degradation under visible light irradiation. The photo-degradation rate of RhB was 94.0~96.0% after only 60 min photocatalytic reaction under visible light irradiation, revealing that it had an excellent visible-light-induced photocatalytic activity. In the fifth recycle, the degradation rate of Ag/BiVO_4_/Mn_1−x_Zn_x_Fe_2_O_4_ still reached to 94.0%. Free radical tunnel experiments confirmed the dominant role of •O_2_^−^ in the photocatalytic process for Ag/BiVO_4_/Mn_1−x_Zn_x_Fe_2_O_4_. Most importantly, the mechanism that multifunction Ag could enhance photocatalytic activity was explained in detail.

## 1. Introduction

Semiconductor photocatalysts have been paid more attention in the application of research of clean energy exploration and environmental pollutants removal. These photocatalysts possess superior physicochemical and magneto-optical properties [1,2,3,4]. Non-toxic bismuth vanadate (BiVO_4_), with good light absorption and high ionic conductivity [5,6,7,8], has attracted strong interest from scientists. Although the visible-light sensitivity and photocatalytic activity of monoclinic scheelite BiVO_4_ (m-BiVO_4_) are the largest among three crystals including additionally tetragonal zircon and tetragonal scheelite [9,10,11,12], the light absorption and the catalytic property of m-BiVO_4_ can be further improved by various strategies. The photocatalytic activity can be greatly improved when the photo-generated electrons and holes are efficiently separated. BiVO_4_-based composites with a high separation efficiency of photo-generated electrons and holes have been developed to enlarge the quantum efficiency of BiVO_4_ and the photocatalytic activity of BiVO_4_. Meanwhile, doping noble metal in photocatalysts is an effective way of promoting the efficient separation of photo-generated electrons and holes. Researchers have reported that the electron–hole separation in doping compounds was strengthened by the charge transfer between semiconductor and noble metal [13,14,15,16,17]. Ag is the most promising noble metal because of the low cost and strong electron trapping ability. Ag-doped catalyst could induce surface plasmon resonance, involving in a better absorption of visible light [18]. 

For the convenience of recovery and separation of photocatalysts after reaction, magnetic photocatalysts have been fabricated in recent years [19,20,21,22]. Magnetic photocatalysts could be recovered with an external magnetic field, and a high recovery ratio would be conducive to promote their industrial application. In our previous research, both the soft-magnetic Mn_1−x_Zn_x_Fe_2_O_4_/Bi_2_O_3_ [21] and hard-magnetic SrFe_12_O_19_/BiVO_4_ [22] with photocatalytic properties were prepared with dip-calcination method. Further exploration is necessary to synthesize m-BiVO_4_-based composite with high photocatalytic activity as well as large recovery ratio. In this work, Ag was doped in BiVO_4_/Mn_1−x_Zn_x_Fe_2_O_4_ with in situ synthesis method. The photocatalytic activity of Ag/BiVO_4_/Mn_1−x_Zn_x_Fe_2_O_4_ was investigated under sunlight irradiation. Further insights are focused on characteristic structure, magnetic property, and photocatalytic mechanism of Ag/BiVO_4_/Mn_1−x_Zn_x_Fe_2_O_4_. 

In fact, fabrication of Ag/BiVO_4_/Mn_1−x_Zn_x_Fe_2_O_4_ was a continuation of our research about the syntheses and application of BiVO_4_/Mn_1−x_Zn_x_Fe_2_O_4_ [19]. The RhB degradation reaction using BiVO_4_/Mn_1−x_Zn_x_Fe_2_O_4_ as photocatalyst was very slow (take 3 h). The incorporation of Ag could enhance the photocatalytic activity of BiVO_4_/Mn_1−x_Zn_x_Fe_2_O_4_. 

## 2. Experimental

### 2.1. Synthesis of Ag/BiVO_4_/Mn_1−x_Zn_x_Fe_2_O_4_

Mn_1−x_Zn_x_Fe_2_O_4_ was prepared according to the literature [19,21].

The precursor of BiVO_4_ was produced by the chemical co-precipitation way [22].

486 mg Mn_1−x_Zn_x_Fe_2_O_4_ was dispersed into the precursor of BiVO_4_ and dried at 80 °C for 12 h. BiVO_4_/Mn_1−x_Zn_x_Fe_2_O_4_ (15.0 wt %) (marked BiVO_4_/Mn_1−x_Zn_x_Fe_2_O_4_) was gained after roasting at 450 °C for 3 h. After dip-roasting, 600 mg BiVO_4_/Mn_1−x_Zn_x_Fe_2_O_4_ was put into 50 mL AgNO_3_ solution (10 mmol/L) under stirring conditions at room temperature for 2 h to form dispersion solution A. 1.0 g polyvinylpyrrolidone (PVP) was added into 50 mL ethanol to obtain the solution B. The dispersion solution A mixed with the solution B. Then the mix solution was heated in water-bath at 70 °C for 4 h. The as-formed mixture was filtered, and washed with water and ethanol, respectively. 12.0 wt % Ag/BiVO_4_/ 15.0 wt % Mn_1−x_Zn_x_Fe_2_O_4_ (marked Ag/BiVO_4_/Mn_1−x_Zn_x_Fe_2_O_4_) was obtained after the filtration residues was dried at 50 °C for 12 h.

### 2.2. Material Characterization

The structure of samples was determined by X-ray diffractometer (Shimadzu, XRD-6000, Kyoto, Japan), Fourier transform infrared spectroscopy (FTIR, Perkin-Elmersystem 2000, Perkin Elmer, Waltham, MA, USA). The ultraviolet-visible diffuse reflectance spectrophotometer (UV-vis DRS, TU1901, Beijing Purkinje, Beijing, China) was employed to examine the light absorption performance of the as-obtained composites. Their morphologies were observed by transmission electron microscopy (TEM, FEI, Tecnai G2 F20, Hillsboro, OR, USA). The element content was analyzed by X-ray photoelectron spectrometer (XPS-XSAM800, Kratos, Manchester, UK) with a base pressure 2 × 10^−7^ Pa and X-ray gun180 W (12 kV, 15 mA). The magnetic properties were investigated using a vibrating sample magnetometer (VSM, Lakeshore 7410, LakeShore, Carson, CA, USA).

### 2.3. Measurement of the Photocatalytic Performance

The photocatalytic activity of the as-prepared composites was investigated by the photodegradation of simulated dye wastewater (Rhodamine B, RhB) under visible light irradiation. 100 mg photocatalyst was put into 100 mL RhB solution of 5 mg/L. Then the suspension liquid was placed in dark for 0.5 h with stirring to reach the adsorption–desorption equilibrium. A 500 W Xe lamp, equipping with UV cut-off filter, was used as the visible light source (λ ≥ 420 nm). At the given irradiation time intervals, a series of the reaction solution was sampled and measured the absorption with the UV-vis spectrophotometer (TU-1901). The photocatalytic mechanism of Ag/BiVO_4_/Mn_1−x_Zn_x_Fe_2_O_4_ was explored by holes-radical trapping experiments with p-benzoquinone (BZQ, •O_2_^−^ radical scavenger), EDTA-Na_2_ (hole scavenger), and tert-butanol (t-BuOH, •OH radical scavenger).

The repeatability of the photocatalyst was detected by cycling tests. After each cycle, the catalyst was separated by an external magnetic field, then washed and dried for the next cycle. 

## 3. Results and Discussion

### 3.1. Synthesis Condition and Structure Identification

The appropriate mass ratio of Mn_1−x_Zn_x_Fe_2_O_4_ and BiVO_4_ was essential for BiVO_4_/Mn_1−x_Zn_x_Fe_2_O_4_. Thus, the composite possessed not only a good magnetization but also a high photocatalytic activity. By the comparison experiments, it is found that the composite held the largest magnetic property without the reduction of photocatalytic activity when 15.0 wt % Mn_1−x_Zn_x_Fe_2_O_4_ was loaded in BiVO_4_ by the dip-calcination approach. The doping quantity of Ag was not only closely related to the photocatalytic activity but also affected the cost of Ag/BiVO_4_/Mn_1−x_Zn_x_Fe_2_O_4_. PVP was confirmed as an efficient stabilizer and reductant in the synthesis process of Ag/BiVO_4_/Mn_1−x_Zn_x_Fe_2_O_4_. Its suitable dosage was 1.0 g in 50 mL ethanol solution. With a series of tests, the optimized doping dosage of Ag in the magnetic composite was determined to be 12.0 wt %. 

**Figure 1:**
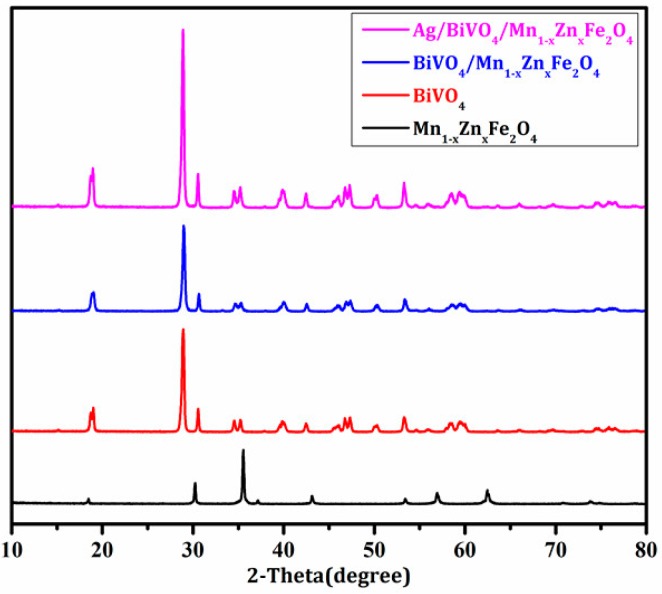
XRD patterns of Mn_1−x_Zn_x_Fe_2_O_4_, BiVO_4_, BiVO_4_/Mn_1−x_Zn_x_Fe_2_O_4_, and Ag/BiVO_4_/Mn_1−x_Zn_x_Fe_2_O_4._

**Figure 2:**
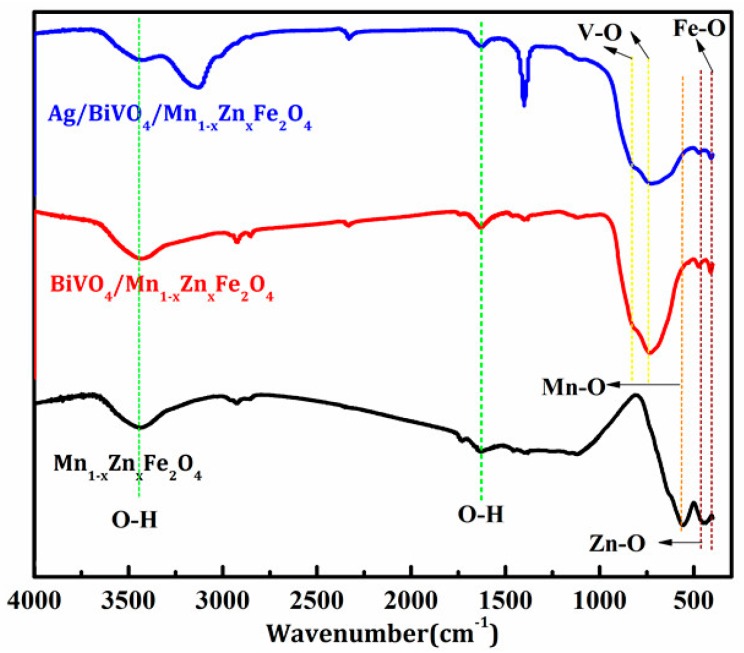
FTIR spectra of Mn_1−x_Zn_x_Fe_2_O_4_, BiVO_4_/Mn_1−x_Zn_x_Fe_2_O_4_, and Ag/BiVO_4_/Mn_1−x_Zn_x_Fe_2_O_4._

XRD patterns of Mn_1−x_Zn_x_Fe_2_O_4_, BiVO_4_, BiVO_4_/Mn_1−x_Zn_x_Fe_2_O_4_, and Ag/BiVO_4_/Mn_1−x_Zn_x_Fe_2_O_4_ were illustrated in Figure 1. It was noticed that each diffraction peak of Mn_1−x_Zn_x_Fe_2_O_4_ was indexed to the franklinite (cubic spinel) phase [23] which belonged to the Fd-3m (227) space group with a lattice size of 0.8474 nm. Three peaks at 28.9°, 35.2°, and 46.0° were clearly attributed to the iconic twin peaks of monoclinic scheelite BiVO_4_ (JCPDS 14-0688) [9]. The lattice parameters of the prepared BiVO_4_ was a = 5.1175 nm, b = 11.6697 nm, and c = 5.1084 nm. The peak at 28.9° (121) was used to calculate the average crystallite size that was 27.2 nm, while the average size of BiVO_4_/Mn_1−x_Zn_x_Fe_2_O_4_ and Ag/BiVO_4_/Mn_1−x_Zn_x_Fe_2_O_4_ was 29.0 nm and 32.8 nm, respectively. To gain further insight into the structure of Ag/BiVO_4_/Mn_1−x_Zn_x_Fe_2_O_4_, we carried out the measurement of bonds vibration absorption with Fourier transform infrared spectroscopy. Figure 2 showed the FTIR spectra of the composites. The vibration peaks of Mn-O, Zn-O, and Fe-O bands of Mn_1−x_Zn_x_Fe_2_O_4_ were severally at 560.1 cm^−1^, 473.7 cm^−1^, and 412.4 cm^−1^, while the V-O vibration absorption peaks of BiVO_4_ was at 734.3 cm^−1^ and 823.4 cm^−1^. This result confirmed the coexistence of Mn_1−x_Zn_x_Fe_2_O_4_ and BiVO_4_ in the composites. The absorption peaks at 2341.7 cm^−1^ and 3433.6 cm^−1^ were ascribed to CO_2_ and the surface adsorption H_2_O. There were not observable characteristic peaks of Ag in Figure 1 and Figure 2 due to its low content [24].

**Figure 3:**
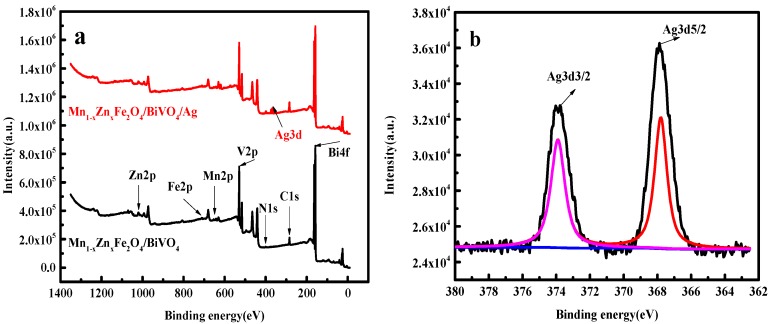

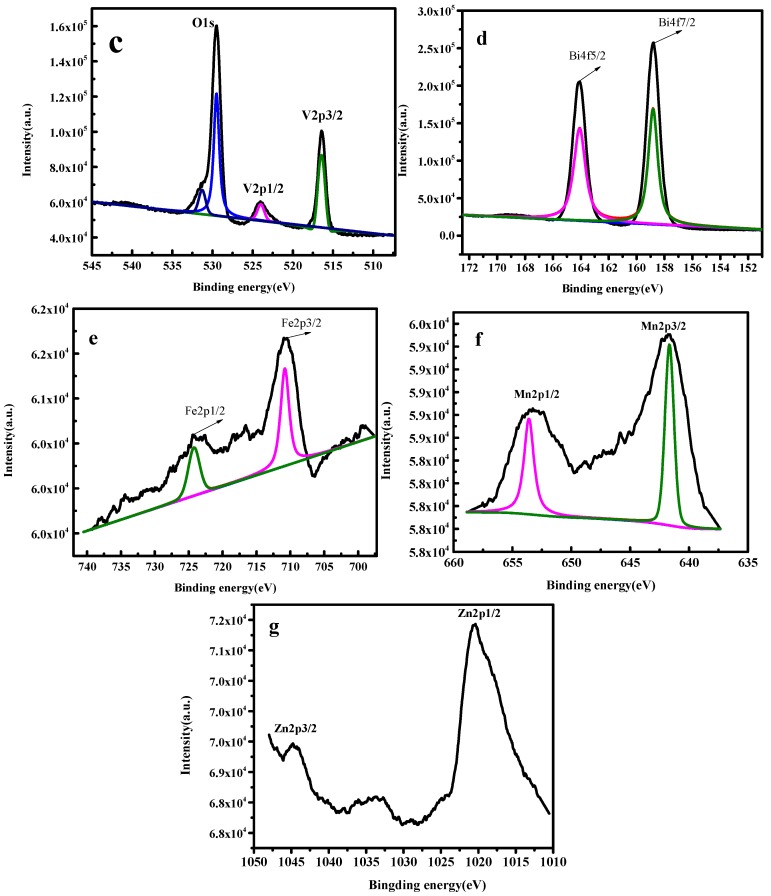
XPS spectra of the magnetic composite (**a**) fully scanned spectra of BiVO_4_/Mn_1−x_Zn_x_Fe_2_O_4_ and Ag/BiVO_4_/Mn_1−x_Zn_x_Fe_2_O_4_; (**b**–**g**) narrow scan spectrum of Ag3d, O1s and V2p, Bi4f, Fe2p, Mn2p, and Zn2p of Ag/BiVO_4_/Mn_1−x_Zn_x_Fe_2_O_4._

To discern the element contents in Ag/BiVO_4_/Mn_1−x_Zn_x_Fe_2_O_4_ and determine their valence states, XPS study was carried out. The binding energy peaks of Ag, O, V, Bi, Fe, Zn, and Mn were recorded in Figure 3. The peaks of O, V, Bi, Fe, Mn, and Zn elements were clearly observed in Figure 3a. Further comparing the fully scanning XPS spectra, it can be seen that the characteristic profile of Ag3d was obvious in Ag/BiVO_4_/Mn_1−x_Zn_x_Fe_2_O_4_ while Ag peak in BiVO_4_/Mn_1−x_Zn_x_Fe_2_O_4_ was not observed. Thus, it was deduced that the doping Ag in BiVO_4_/Mn_1−x_Zn_x_Fe_2_O_4_ was successful [25]. The peaks at the binding energy of 373.9 eV and 367.9 eV in Figure 3b was severally ascribed to Ag 3d_3/2_ and 3d_5/2_ [19], revealing the existence of Ag^+^. In Figure 3c, the peaks of O1s, V2p_3/2_, and V2p_1/2_ were located at 530.5 eV, 516.5 eV, and 523.4 eV, which were assigned to O_2_^−^ and V-O bands. There were peaks of Bi4f_5/2_ and 4f_7/2_ at 164.1 eV and 158.3 eV in Figure 3d, indicating the presence of bismuth species of Bi^3+^ in BiVO_4_. Figure 3f displayed peaks at the binding energy of 641.5 eV (Mn2p_3/2_) and 653.1 eV (Mn2p_1/2_). The high resolution spectra of Fe2p as well as Zn2p were shown in Figure 3e,g. These peaks verified the presence of Mn_1−x_Zn_x_Fe_2_O_4_, which was consistent with the results of XRD and FTIR detection [26]. So, Ag/BiVO_4_/Mn_1−x_Zn_x_Fe_2_O_4_ was successfully assembled by in situ wet-chemistry synthesis method. This synthesis approach was simple, low cost, and environmentally friendly.

**Figure 4:**
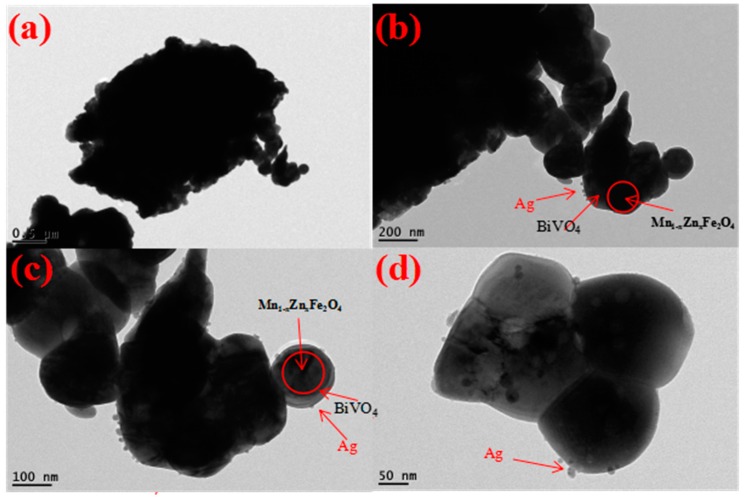
TEM images of Ag/BiVO_4_/Mn_1−x_Zn_x_Fe_2_O_4._

The morphological analysis of Ag/BiVO_4_/Mn_1−x_Zn_x_Fe_2_O_4_ was studied with transmission electron microscopy (TEM), and the results were displayed in Figure 4. By comparative experiments, it was demonstrated that the surface property of Ag/BiVO_4_/Mn_1−x_Zn_x_Fe_2_O_4_ was significantly improved when the appropriate dosage of polyvinylpyrrolidone (PVP) was used in the fabrication process of the composite. The improvement in properties resulted from the surface activity of PVP and the full uniform dispersion of Ag ions in the reaction solution. In addition, ethanol (solvent) further promoted the complete interface reaction of the ions with BiVO_4_/Mn_1−x_Zn_x_Fe_2_O_4_ particles. Namely, PVP could prompt the formation of nano-structural particles through in situ wet-chemistry method.

It was noted in Figure 4a,b that the bright surface sphere of BiVO_4_ attached the dark particles of Mn_1−x_Zn_x_Fe_2_O_4_. A small amount of Ag granular particles uniformly dispersed in the spherical surface in Figure 4c,d. As estimated from the images of BiVO_4_/Mn_1−x_Zn_x_Fe_2_O_4_, the average size of Ag granular particles was about 30 nm. The granular nanostructure particle of Ag favored production of rich active sites in the photocatalyst.

### 3.2. Light Absorption Property and Magnetic Property

**Figure 5:**
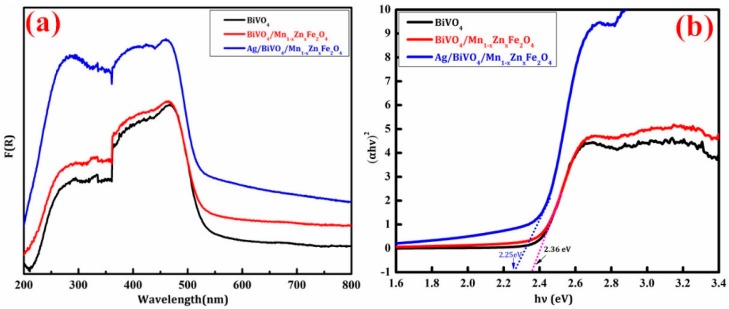
(**a**) UV-vis diffuse reflection spectra of products and (**b**) plot of (Ahυ)^2^ versus photon energy (hυ) according to the UV-vis DRS.

UV-vis diffuse reflectance spectrophotometry was a suitable and important technique to determine the light absorption for semiconductor photocatalysts [27]. Figure 5 showed UV-vis diffuse reflectance spectra (UV-vis DRS) of BiVO_4_, BiVO_4_/Mn_1−x_Zn_x_Fe_2_O_4_, and Ag/BiVO_4_/Mn_1−x_Zn_x_Fe_2_O_4_. It can be discovered from Figure 5a that the maximum absorption edge of Ag/BiVO_4_/Mn_1−x_Zn_x_Fe_2_O_4_ shifted to red light region, leading to the main absorption edge around 400 nm. First, the red shift was directly related to the electrons and Ag^+^ transformation between the conduction band and the valence band of BiVO_4_ [17]. Second, Ag particles had darkened color to enhance absorption of the visible-light for BiVO_4_/Mn_1−x_Zn_x_Fe_2_O_4_. Third, Ag particles could produce a strong surface plasmon resonance absorption. The band-gap energy (E_g_) for Ag/BiVO_4_/Mn_1−x_Zn_x_Fe_2_O_4_ was about 2.25 eV. E_g_ values of BiVO_4_ and BiVO_4_/Mn_1−x_Zn_x_Fe_2_O_4_ in Figure 4b were about 2.36 eV. The incorporation of Mn_1−x_Zn_x_Fe_2_O_4_ did not change the optical properties of BiVO_4_ [18]. The relatively low E_g_ of Ag/BiVO_4_/Mn_1−x_Zn_x_Fe_2_O_4_ appeared to strengthen the absorption and sensitivity response for visible light. The significant enhancement of optics properties would be conducive to bringing high photocatalytic activity.

**Figure 6:**
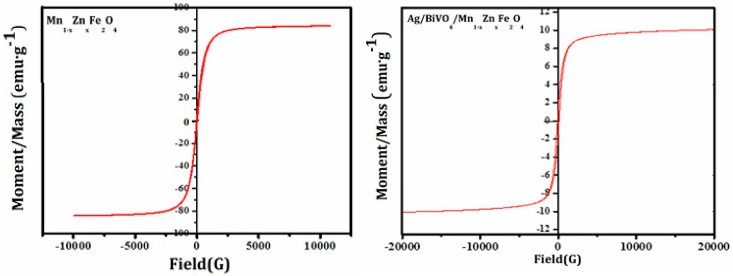
Hysteresis loops of BiVO_4_/Mn_1−x_Zn_x_Fe_2_O_4_ and Ag/BiVO_4_/Mn_1−x_Zn_x_Fe_2_O_4_.

Hysteresis loops are the key way to characterize magnetization for magnetic materials. Figure 6 recorded the hysteresis loops of Ag/BiVO_4_/Mn_1−x_Zn_x_Fe_2_O_4_ and pure Mn_1−x_Zn_x_Fe_2_O_4_. The saturation magnetization (Ms) of Ag/BiVO_4_/Mn_1−x_Zn_x_Fe_2_O_4_ in Figure 6 was 10.04 emu/g. It was noted that large Ms was conducive towards the separation and recovery with an external magnet. Compared with Mn_1−x_Zn_x_Fe_2_O_4_, the Ms of Ag/BiVO_4_/Mn_1−x_Zn_x_Fe_2_O_4_ was declined by 87.5% due to the decrease of magnetic content per unit mass. More importantly, Ms of Ag/BiVO_4_/Mn_1−x_Zn_x_Fe_2_O_4_ was larger than that (7.01 emu/g) of Mn_1−x_Zn_x_Fe_2_O_4_/Bi_2_O_3_ [21]. The magnetic property was conducive to the stable activity of Ag/BiVO_4_/Mn_1−x_Zn_x_Fe_2_O_4_. The result revealed that the as-prepared magnetic photocatalyst was easily recovered by an external magnet. Therefore, it was concluded that Ag/BiVO_4_/Mn_1−x_Zn_x_Fe_2_O_4_ with good magnetic property possessed a high recovery rate. 

### 3.3. Photocatalytic Activity

It was well-known that the photocatalytic ability was vital to photocatalytic materials, which was the base property for their industrial application. Generally, the photocatalytic activity was assessed with the degradation reaction of dyes. 

#### 3.3.1. Visible-Light-Driven Photocatalytic Activity

**Figure 7:**
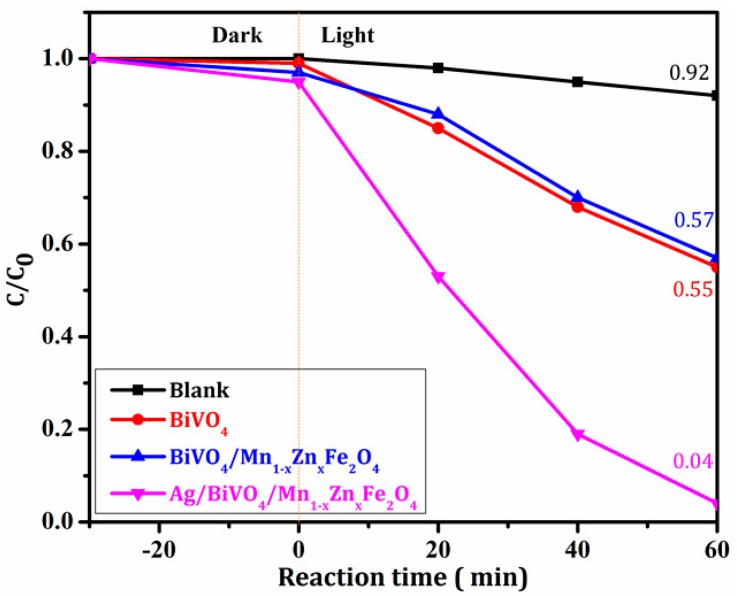
The degradation rates of RhB with the three photocatalysts.

The photocatalytic performance of the samples under visible light irradiation was evaluated with the RhB photodegradation, and the result was shown in Figure 7. There was only a little degradation rate in the blank test (without any photocatalyst), indicating the poor self-degradation of RhB. The degradation rate for BiVO_4_ and BiVO_4_/Mn_1−x_Zn_x_Fe_2_O_4_ was approximately 45.0% after 60 min reaction. The same degradation rate proved that the introduction of Mn_1−x_Zn_x_Fe_2_O_4_ did not cause the activity loss of BiVO_4_. Figure 7 indicates that the degradation rate for Ag/BiVO_4_/Mn_1−x_Zn_x_Fe_2_O_4_ reached to 96.0% under the same condition. The setting time was only 60 min in this photodegradation test of RhB. Hence, the photocatalytic property of Ag/BiVO_4_/Mn_1−x_Zn_x_Fe_2_O_4_ was obviously higher than that of BiVO_4_ and BiVO_4_/Mn_1−x_Zn_x_Fe_2_O_4_. It meant that only 12.0 wt % Ag brought outstanding improvement in photocatalytic ability of BiVO_4_/Mn_1−x_Zn_x_Fe_2_O_4_. 

In fact, most degradation tests are very slow (may take several hours) despite the improvements in visible light absorption of the photocatalyst. Here, the as-prepared Ag/BiVO_4_/Mn_1−x_Zn_x_Fe_2_O_4_ has a highly photocatalytic efficiency. This can be explained with the following three aspects: (1) Ag produced the surface plasmon resonance (plasma energy), which was transferred to BiVO_4_, leading to more formation of photo-excited electrons and holes. It was helpful to the enhancement of photocatalytic activity; (2) Ag particles acted as holes and accepted photo-produced electrons from the conduction band of BiVO_4_, extending the wavelength range and preventing the recombination of electrons and holes. The transformation or conversion of the charged particles in the interface was strengthened. In other words, the presence of Ag particles boosted the quantum efficiency for BiVO_4_/Mn_1−x_Zn_x_Fe_2_O_4_; (3) Owing to the nanostructure of Ag particles, Ag/BiVO_4_/Mn_1−x_Zn_x_Fe_2_O_4_ possessed a relatively large specific surface area, which increased the efficient sites and further yielded a high photocatalytic activity [19,25]. Thus, nanostructure Ag particles ensured high photocatalytic property of Ag/BiVO_4_/Mn_1−x_Zn_x_Fe_2_O_4_. 

Ag/BiVO_4_/Mn_1−x_Zn_x_Fe_2_O_4_ was recovered with an external magnet in the end of the photocatalytic degradation test. 88~91 mg (after washing and drying) Ag/BiVO_4_/Mn_1−x_Zn_x_Fe_2_O_4_ could recover from initial dosage of 100 mg in each cycle. The average recovery rate of magnetic Ag/BiVO_4_/Mn_1−x_Zn_x_Fe_2_O_4_ was 89%, which was larger than the literature report value (85.0%) [28]. It is worth mentioning that the recovery method was quick with low energy consumption. The high recovery rate effectively avoided the leftover of catalysts in the water solution. Namely, Ag/BiVO_4_/Mn_1−x_Zn_x_Fe_2_O_4_ demonstrated itself as an environmentally friendly photocatalytic material and showed perspective industrial application in removal water-soluble contaminants.

**Figure 8:**
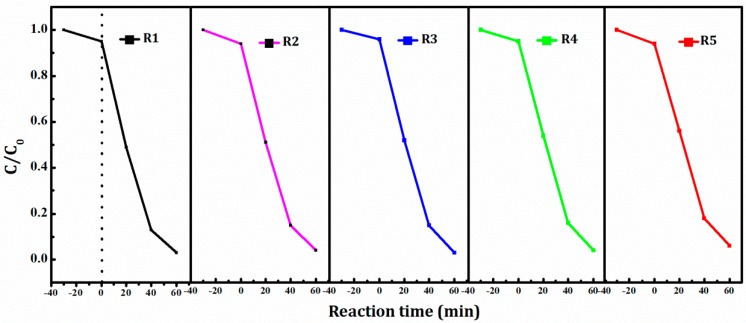
Cycling tests of Ag/BiVO_4_/Mn_1−x_Zn_x_Fe_2_O_4_.

The repeatability and stability were necessary in the practical photocatalytic application [27]. Cycling tests were employed to evaluate the photocatalytic stability of Ag/BiVO_4_/Mn_1−x_Zn_x_Fe_2_O_4_, and the degradation rate of RhB was described in Figure 8. It was clear that the degradation rate during the five cycles was severally 96.0%, 96.0%, 95.0%, 94.0% and 94.0%, which revealed photocatalyst efficiency of Ag/BiVO_4_/Mn_1−x_Zn_x_Fe_2_O_4_ barely decreased in the test process. Experiment results exhibited excellent photocatalytic stability. What is more, 94.0% of the degradation rate during the five cycles was very larger than that of the reference report [13]. So, magnetic photocatalyst Ag/BiVO_4_/Mn_1−x_Zn_x_Fe_2_O_4_ possessed promising prospect in the photo-decomposition organic dyes (industrial wastewater) field.

#### 3.3.2. Photocatalytic Mechanism

**Figure 9:**
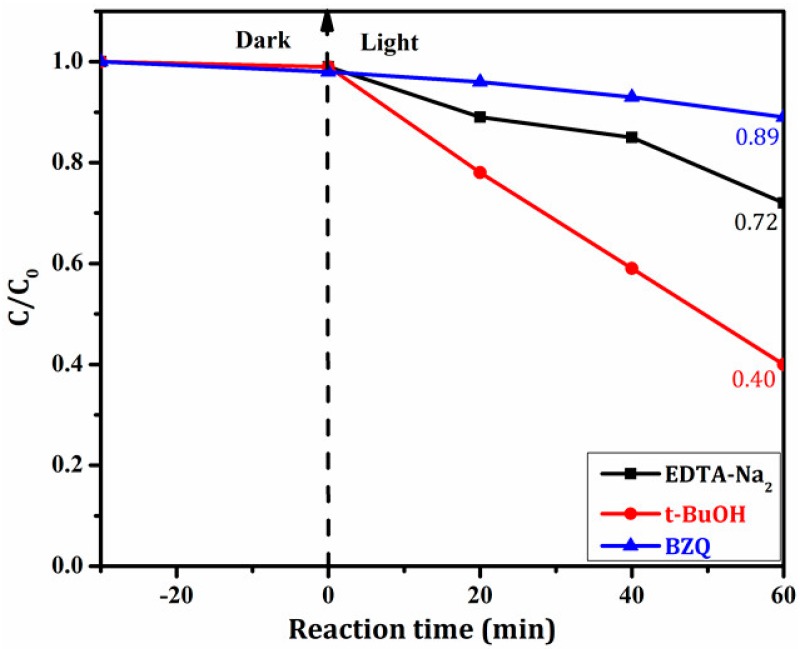
The photodegradation rate of RhB with Ag/BiVO_4_/Mn_1−x_Zn_x_Fe_2_O_4_ and different scavengers.

Radical scavengers were used to study active species in photocatalytic reaction, the result was displayed in Figure 9. In details, the degradation rate of RhB in Ag/BiVO_4_/Mn_1−x_Zn_x_Fe_2_O_4_-EDTA-Na_2_ (h^+^ scavenger) lowed and reached to 28.0%, which was significantly lower than that of Ag/BiVO_4_/Mn_1−x_Zn_x_Fe_2_O_4_. Under the same condition, the degradation rate steeply went down when BZQ (•O_2_^−^ scavenger) in place of EDTA-Na_2_ was added into the reaction solution, the rate was only 11.0%. However, the introduction of t-BuOH (•OH scavenger) caused a large decrease in the degradation of about 60.0%. Namely, the change of the degradation rate in Ag/BiVO_4_/Mn_1−x_Zn_x_Fe_2_O_4_-t-BuOH was the smallest among three radical scavenger tests. The results illustrated that free radicals were major active species, and that •O_2_^−^ played the domination role though •OH and h^+^ took part in the photocatalytic reaction. 

**Figure 10:**
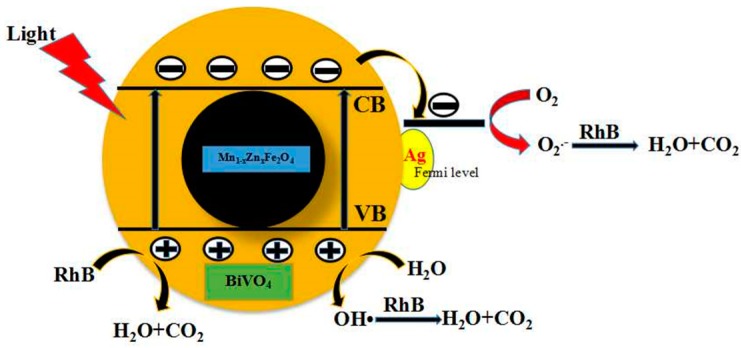
Photocatalytic mechanism scheme of Ag/BiVO_4_/Mn_1−x_Zn_x_Fe_2_O_4_.

The electron transition occurred between valence band and conduction band, generating the photoelectrons and holes when the photon energy was higher than E_g_ of the semiconductor. The possible transition of photo-induced electron and hole was used to express the photocatalytic process under light irradiation. The photocatalytic mechanism of Ag/BiVO_4_/Mn_1−x_Zn_x_Fe_2_O_4_ was described in Figure 10. In detail, e^-^ transferred to the surface of Ag particle, the dissolved oxygen (O_2_) could capture the electron and form the super oxygen radical (•O_2_^−^ ) through the Fermi level surface resonance. The adsorbed H_2_O in the surface of Ag/BiVO_4_/Mn_1−x_Zn_x_Fe_2_O_4_ could be oxidized by holes (h^+^), yielding hydroxyl free radical (•OH). Both •O_2_^−^ and •OH had a large oxidation ability and decomposed RhB into CO_2_ and H_2_O. At the same time, holes themselves prompted the degradation-oxidized of RhB [26]. Thus, the doping Ag was favorable to drive more •O_2_^−^ and •OH radicals, strengthening the degradation of RhB in visible light irradiation.

In fact, we used Mn_1−x_Zn_x_Fe_2_O_4_ as magnetic substrate in order to simplify separation after photocatalytic reaction for BiVO_4_. The UV-vis DRS shown the incorporation of Mn_1−x_Zn_x_Fe_2_O_4_ did not enhance the optical properties of BiVO_4_. Noble metal-doping and graphene-loading were good ways to improve optical properties and enhance photocatalytic activity for single phase semiconductor. Here, we chose Ag-doping to boost the photocatalytic activity of BiVO_4_/Mn_1−x_Zn_x_Fe_2_O_4_. In addition, we will use graphene to modify BiVO_4_/Mn_1−x_Zn_x_Fe_2_O_4_. These studies will help to choose a better way (the above mentioned) for enhancing photocatalytic activity via comparing their photocatalytic activity and reaction kinetics, and then apply these findings to other signal semiconductors. 

## 4. Conclusions

Ag/BiVO_4_/Mn_1−x_Zn_x_Fe_2_O_4_ was fabricated with the dip-calcination and in situ wet-chemistry synthesis method that was simple and environmentally-friendly. Element contents and their valence states in Ag/BiVO_4_/Mn_1−x_Zn_x_Fe_2_O_4_ were detected, indicating Ag granular particles dispersed in the spherical surface of BiVO_4_. The presence of Ag particles boosted the quantum efficiency, and further enhanced the photocatalytic activity. Under visible light irradiation (λ ≥ 400nm), the degradation rate of RhB using Ag/BiVO_4_/Mn_1−x_Zn_x_Fe_2_O_4_ after only 60 min reaction reached to 96.0%, which was greater than that of Mn_1−x_Zn_x_Fe_2_O_4_/BiVO_4_ and pure BiVO_4_. Most importantly, the degradation rate was close to 94.0% during the fifth recycle. We hope this research can promote the industrial application of BiVO_4_.

## References

[1] Yu J.Q., Kudo A. (2005). Hydrothermal synthesis of nanofibrous bismuth vanadate. J. Chem. Lett..

[2] Xia D.H., Hua L.L., Tan X.Q., He C., Pan W.Q., Yang T.S., Huang Y.L., Shu D. (2016). Immobilization of self-stabilized plasmonic Ag-AgI on mesoporous Al_2_O_3_ for efficient purification of industrial waste gas with indoor LED illumination. Appl. Catal. B: Environ..

[3] Zhang L.S., Lian J.S., Wu L.Y., Duan Z.R., Jiang J., Zhao L.J. (2014). Synthesis of a Thin-Layer MnO_2_ Nanosheet-Coated Fe_3_O_4_ Nanocomposite as a Magnetically Separable Photocatalyst. Langmuir.

[4] Aiga N., Jia Q.X., Watanabe K., Kudo A., Sugimoto T., Matsumoto Y. (2013). Electron–Phonon Coupling Dynamics at Oxygen Evolution Sites of Visible-Light-Driven Photocatalyst: Bismuth Vanadate. J. Phys. Chem. C.

[5] Chang X.X., Wang T., Zhang P., Zhang J.J., Li A., Gong J.L. (2015). Enhanced Surface Reaction Kinetics and Charge Separation of p–n Heterojunction Co_3_O_4_/BiVO_4_ Photoanodes. J. Am. Chem. Soc..

[6] Seabold J.A., Choi K.S. (2012). Efficient and Stable Photo-Oxidation of Water by a Bismuth Vanadate Photoanode Coupled with an Iron Oxyhydroxide Oxygen Evolution Catalyst. J. Am. Chem. Soc..

[7] Li H.Y., Sun Y.J., Cai B., Gan S.Y., Han D.X., Niu L., Wu T.S. (2015). Hierarchically Z-scheme photocatalyst of Ag@AgCl decorated on BiVO_4_(040) with enhancing photoelectrochemical and photocatalytic performance. Appl. Catal. B Environ..

[8] Wang W.Z., Huang X.W., Wu S., Zhou Y.I., Wang L.J., Shi H.L., Liang Y.J., Zou B. (2013). Preparation of p–n junction Cu_2_O/BiVO_4_ heterogeneous nanostructures with enhanced visible-light photocatalytic activity. Appl. Catal. B Environ..

[9] Wang A.L., Shen S., Zhao Y.B., Wu W. (2015). Preparation and characterizations of BiVO_4_/reduced graphene oxide nanocomposites with higher visible light reduction activities. J. Colloid Intel. Sci..

[10] Yu Q.Q., Tang Z.R., Xu Y.J. (2014). Synthesis of BiVO_4_ nanosheets-graphene composites toward improved visible light photoactivity. J. Energy Chem..

[11] Abdi F.F., Dabirian A., Dam B., van de Krol R. (2014). Plasmonic enhancement of the optical absorption and catalytic efficiency of BiVO_4_ photoanodes decorated with Ag@SiO_2_ core-shell nanoparticles. Phys. Chem. Chem. Phys..

[12] Li R.G., Han H.X., Zhang F.X., Wang D., Li C. (2014). Highly efficient photocatalysts constructed by rational assembly of dual-cocatalysts separately on different facets of BiVO_4_. Energy Environ. Sci..

[13] Xu L., Wei Y.G., Guo W., Guo Y.H., Guo Y.N. (2015). One-pot solvothermal preparation and enhanced photocatalyticactivity of metallic silver and graphene co-doped BiVO4ternarysystems. Appl. Surf. Sci..

[14] Rismanchian A., Chen Y.W., Chuang S.S.C. (2016). In situ infrared study of photoreaction of ethanol on Au and Ag/TiO_2_. Catal. Today.

[15] Benedetti J.E., Bernardo D.R., Morais A., Bettini J., Nogueira A.F. (2015). Synthesis and characterization of a quaternary nanocomposite based on TiO_2_/CdS/rGO/Pt and its application in the photoreduction of CO_2_ to methane under visible light. RSC Adv..

[16] Xue Y., Wang X.T. (2015). The effects of Ag doping on crystalline structure and photocatalytic properties of BiVO_4_. Int. J. Hydrogen Energy.

[17] Chen L., Huang R., Ma Y.J., Luo S.L., Au C.T., Yin S.F. (2013). Controllable synthesis of hollow and porous Ag/BiVO_4_ composites with enhanced visible-light photocatalytic performance. RSC Adv..

[18] Chen F., Yang Q., Wang Y.L., Zhao J.W., Wang D.B., Li X.M., Guo Z., Wang H., Deng Y.C., Niu C.G. (2017). Novel ternary heterojunction photcocatalyst of Ag nanoparticles and g-C_3_N_4_ nanosheets co-modified BiVO_4_ for wider spectrum visible-light photocatalytic degradation of refractory pollutant. Appl. Catal. B Environ..

[19] Xie T.P., Liu C.L., Xu L.J., Li H. (2018). New Insights into Mn_x_Zn_1−x_Fe_2_O_4_ via Fabricating Magnetic Photocatalyst Material BiVO_4_/Mn_x_Zn_1−x_Fe_2_O_4_. Materials.

[20] Chi Y., Yuan Q., Li Y.J., Zhao L., Li N., Li X.T., Yan W.F. (2013). Magnetically separable Fe_3_O_4_@SiO_2_@TiO_2_-Ag microspheres withwell-designed nanostructure and enhanced photocatalytic. J. Hazard. Mater..

[21] Zhang Z.D., Xu L.J., Liu C.L. (2015). Preparation and characterization of composite magnetic photocatalyst Mn_x_Zn_1−x_Fe_2_O_4_/β-Bi_2_O_3_. RSC Adv..

[22] Liu C.L., Li H., Ye H.P., Xu L.J. (2015). Preparation and Visible-Light-Driven Photocatalytic Performance of Magnetic SrFe_12_O_19_/BiVO_4_. J. Mater. Eng. Perform..

[23] Laohhasurayotin K., Pookboonmee S., Viboonratanasri D., Kangwansupamonkon W. (2012). Preparation of magnetic photocatalyst nanoparticles TiO_2_/SiO_2_/Mn–Zn ferrite and its photocatalytic activity influenced by silica interlayer. Mater. Res. Bull..

[24] Wu S.K., Shen X.P., Zhu G.X., Zhou H., Ji Z.Y., Chen K.M., Yuan A.H. (2016). Synthesis of ternary Ag/ZnO/ZnFe_2_O_4_ porous and hollownanostructures with enhanced photocatalytic activity. Appl. Catal. B Environ..

[25] Saravanakumar K., Ramjan M.M., Suresh P., Muthuraj V. (2016). Fabrication of highly efficient visible light driven Ag/CeO_2_ photocatalyst for degradation of organic pollutants. J. Alloys Comp..

[26] Xie T.P., Liu C.L., Xu L.J., Yang J., Zhou W. (2013). Novel Heterojunction Bi_2_O_3_/SrFe_12_O_19_ Magnetic Photocatalyst with Highly Enhanced Photocatalytic Activity. J. Phys. Chem. C.

[27] Yang J., Xu L.J., Liu C.L., Xie T.P. (2014). Preparation and photocatalytic activity of porous Bi_5_O_7_I nanosheets. Appl. Surf. Sci..

[28] Xie T.P., Xu L.J., Liu C.L., Yang J., Wang M. (2014). Magnetic composite BiOCl–SrFe_12_O_19_: A novel p-n type heterojunction with enhanced photocatalytic activity. Dalt. Trans..

